# A Structured and Methodological Review on Multi-View Human Activity Recognition for Ambient Assisted Living

**DOI:** 10.3390/jimaging11060182

**Published:** 2025-06-03

**Authors:** Fahmid Al Farid, Ahsanul Bari, Abu Saleh Musa Miah, Sarina Mansor, Jia Uddin, S. Prabha Kumaresan

**Affiliations:** 1 Faculty of Engineering, Multimedia University, Cyberjaya 63100, Malaysia; ahsanulbari99@gmail.com; 2 Department of Computer Science and Engineering, Bangladesh Army University of Science and Technology (BAUST), Saidpur 5311, Bangladesh; musa@baust.edu.bd; 3 AI and Big Data Department, Woosong University, Daejeon 34606, Republic of Korea; jia.uddin@wsu.ac.kr; 4 Centre for Image and Vision Computing (CIVC), COE for Artificial Intelligence, Faculty of Artificial Intelligence and Engineering (FAIE), Multimedia University, Cyberjaya 63100, Malaysia; prabha.kumaresan@mmu.edu.my

**Keywords:** Ambient Assisted Living, lightweight deep learning, activity recognition, machine learning, wearable sensors, smartphones, context-aware, deep learning

## Abstract

Ambient Assisted Living (AAL) leverages technology to support the elderly and individuals with disabilities. A key challenge in these systems is efficient Human Activity Recognition (HAR). However, no study has systematically compared single-view (SV) and multi-view (MV) Human Activity Recognition approaches. This review addresses this gap by analyzing the evolution from single-view to multi-view recognition systems, covering benchmark datasets, feature extraction methods, and classification techniques. We examine how activity recognition systems have transitioned to multi-view architectures using advanced deep learning models optimized for Ambient Assisted Living, thereby improving accuracy and robustness. Furthermore, we explore a wide range of machine learning and deep learning models—including Convolutional Neural Networks (CNNs), Recurrent Neural Networks (RNNs), Long Short-Term Memory (LSTM) networks, Temporal Convolutional Networks (TCNs), and Graph Convolutional Networks (GCNs)—along with lightweight transfer learning methods suitable for environments with limited computational resources. Key challenges such as data remediation, privacy, and generalization are discussed, alongside potential solutions such as sensor fusion and advanced learning strategies. This study offers comprehensive insights into recent advancements and future directions, guiding the development of intelligent, efficient, and privacy-compliant Human Activity Recognition systems for Ambient Assisted Living applications.

## 1. Introduction

Human Activity Recognition (HAR) is a fundamental research area, particularly in Ambient Assisted Living (AAL) environments where safety, independence, and well-being of elderly and disabled people are of major concern. The continuous evolution of sensor technologies, along with the power of deep learning, has revolutionized the area, making it possible to monitor and classify human activities with high accuracy in real time. Multi-view HAR (MV-HAR), which integrates data from multiple sensors, has emerged as a robust solution to overcome the limitations of single-view approaches. This review focuses on the advancements in lightweight deep learning models that optimize HAR systems, allowing them to operate efficiently in resource-constrained AAL environments. By synthesizing recent research, this paper identifies gaps and suggests future directions for enhancing HAR systems’ scalability and computational efficiency.

Deep learning is a subset of machine learning that uses artificial neural networks with multiple layers to automatically learn hierarchical representations of data. Unlike traditional models, deep learning methods eliminate the need for manual feature engineering by extracting features directly from raw input, such as images or sensor signals. This has made deep learning particularly effective in fields like image recognition, speech processing, and activity recognition, where complex patterns need to be identified in large datasets.

### 1.1. Applications in Ambient Assisted Living (AAL)

Precise, versatile HAR solutions are of key relevance to AAL systems. Lightweight deep learning models are particularly well suited to AAL applications. The advantages of MV-HAR are as follows:

- Fall Detection: Fall detection is required to ensure the safety of the elderly living alone.
- Activity Monitoring: Provides a means of monitoring Activities of Daily Living (ADL), which can detect physical and cognitive health changes when there are abnormalities detected in MV-HAR.
- Anomaly Detection: Detection of significant deviations from normal patterns can be reflective of health events, e.g., falls.
- By LIFEBYTES: MV-HAR monitors exercises and guides rehabilitation progress monitoring.

### 1.2. Integration in AAL Systems

The synergy of multi-view HAR and lightweight deep learning models provides an end-to-end solution for developing ubiquitous and real-time Ambient Assisted Living (AAL) systems. This paper presents case studies and applications that have been favorably affected using this synergistic framework as evidenced through high inference accuracy for daily activity and system performance [1]. The potential of such technologies for assisting daily activity and emergent event detection (e.g., falls and medical events) is particularly highlighted.

### 1.3. Multi-View Human Activity Recognition

To counter the limitation of single-view Human Activity Recognition systems, one of the solutions is MV-HAR. MV-HAR uses multiple synchronized high-resolution wide-angle cameras of various views to fuse information from multiple views to provide a complete and detailed description of complex activities. Even though hand-crafted features dominated early research of MV-HAR, deep learning-based approaches now prevail over traditional ones.

### 1.4. Research Questions

To guide this study, we have formulated the following primary research questions:

- Research Question 1 (RQ1): How have hand gesture recognition (HGR) systems evolved from 2014 to 2025 in terms of vision-based, sensor-based, and multimodal approaches, particularly focusing on advancements in data acquisition methods, environmental settings, and gesture representation techniques?
- Research Question 2 (RQ2): What has been the effectiveness of current HGR systems employing various data modalities, and what are the emerging trends and future directions in this field?
- Research Question 3 (RQ3): How do the challenges and limitations associated with different HGR modalities (e.g., vision-only vs. multimodal systems) impact system performance, usability, and scalability in real-world applications?

### 1.5. Background

HAR involves using sensors and algorithms to detect and classify human activities based on data streams, such as those generated by accelerometers, gyroscopes, and cameras. This technology is crucial in diverse fields, including healthcare, smart environments, and industrial monitoring. In the context of AAL, HAR plays a vital role in tracking daily activities, predicting health risks, and providing timely assistance to the elderly and individuals with disabilities.

Historically, HAR systems relied on single-view sensors, which posed challenges due to limited perspectives and occlusions. To address these shortcomings, MV-HAR has been introduced, allowing for more comprehensive monitoring by incorporating data from multiple sources, such as fixed and wearable cameras. The challenge lies in the computational complexity of these systems, which impedes their real-time deployment in environments like AAL, where resources are often limited. Lightweight deep learning models, designed for efficiency and accuracy, have shown promise in tackling this challenge by optimizing sensor data processing without compromising performance.

### 1.6. Motivation

The rapid increase in global life expectancy has created a pressing need for technologies that support independent living for the elderly and disabled. AAL systems, powered by HAR, can monitor individuals’ daily activities and provide crucial insights into their well-being, offering a safety net in scenarios like fall detection and health monitoring. However, the high computational demands of traditional HAR systems, particularly when using deep learning models, present significant challenges in deploying these technologies in real-time, resource-constrained environments.

This review is motivated by the need to explore recent advancements in lightweight deep learning models that address these computational challenges. By examining the integration of multi-view data and deep learning techniques, we aim to identify solutions that can enhance the efficiency, scalability, and accuracy of HAR systems in AAL environments. Moreover, this paper highlights the potential for lightweight models, such as Mobile LeNet and Mobile Neural Architecture Search Network (MnasNet), to outperform more complex systems, making them ideal for real-time applications in AAL settings.

In this review, we make the following key contributions to the field of multi-view Human Activity Recognition (MV-HAR) for Ambient Assisted Living (AAL):

1. **Comprehensive Analysis of MV-HAR Methods:** We provide a detailed survey of the state-of-the-art MV-HAR methods, exploring the transition from single-view to multi-view approaches. This includes a comparative evaluation of existing models and a discussion on how multi-view architectures enhance the accuracy and robustness of HAR systems in AAL environments.
2. **Focus on Lightweight Deep Learning Models:** We highlight the advantages of lightweight deep learning architectures tailored for resource-constrained AAL settings. Our review covers novel techniques that balance computational efficiency with accuracy, making them suitable for real-time applications in AAL.
3. **Examination of Key Challenges and Solutions:** We address several pressing challenges in the field, including issues related to data heterogeneity, privacy concerns, and the need for generalizable HAR models. In response, we explore solutions such as sensor fusion, transfer learning, and privacy-preserving techniques that improve the efficacy of HAR systems.
4. **Guideline for Future Research:** By synthesizing the current progress and identifying gaps in the literature, we offer a set of guidelines and future directions aimed at developing more intelligent, scalable, and privacy-compliant AAL systems. This serves as a foundation for researchers looking to innovate in the intersection of MV-HAR and AAL.

The rest of the paper is organized as follows: Section 3 explains the dataset in detail. Section 4 explains the proposed methodology in detail, such as data preprocessing, techniques, positional activity and skeleton extraction, segmentation, and classification approaches. Section 5 provides the experimental analysis, such as performance and accuracy. Section 6 concludes the paper.

## 2. Related Work

Recent research on Human Activity Recognition (HAR) has extensively explored the use of machine learning and deep learning techniques to achieve significant improvements in both accuracy and computational efficiency [2,3,4,5,6,7,8,9,10,11,12,13,14,15]. Deep learning models have revolutionized HAR, particularly through the use of lightweight architectures designed for resource-constrained environments like Ambient Assisted Living (AAL) systems [16,17]. These models, such as Mobile LeNet (M-LeNet) and Mobile Neural Architecture Search Network (MnasNet), offer superior performance in AAL scenarios due to their ability to handle multi-view data from various sensors, including fixed cameras and wearable devices [18]. Several studies have demonstrated the effectiveness of lightweight CNN architectures in improving HAR systems’ computational efficiency and scalability [19]. For instance, the integration of multi-view data collection and lightweight deep learning models has been shown to significantly enhance the ability to monitor activities in AAL environments. Using the Robot Human Monitoring–Human Activity Recognition–Skeleton (RHM-HAR-SK) dataset, lightweight models such as M-LeNet and MnasNet have outperformed more complex models by offering a balance between accuracy and computational resource demands [20]. In addition, HAR research has expanded to include hybrid deep learning models that incorporate both conventional and emerging methods, addressing challenges such as sensor data synchronization and privacy concerns [19,21]. These hybrid models leverage multi-modal data sources, such as accelerometer data, video sequences, and audio signals, which improve HAR system robustness and generalization across different environments and activities [22]. The integration of skeleton information has also been explored to enhance activity recognition, particularly in detecting critical events like falls, which are vital in AAL systems [20]. Other studies have explored the potential of large language models to automate the filtering and taxonomy creation of relevant academic papers in the HAR domain, which has accelerated the pace of research [19]. These taxonomies categorize deep learning models into two main types: conventional models, which rely on traditional feature extraction techniques, and hybrid models, which combine multiple modalities to enhance HAR system performance [17]. This approach has allowed researchers to identify gaps in the literature, particularly in terms of data diversity, privacy preservation, and computational limitations in real-time applications [23]. Furthermore, the rise in multimodal HAR systems, which integrate information from various sensor types, including video and inertial data, have proven effective in increasing the accuracy and applicability of HAR in real-world scenarios. For example, studies using the UP-Fall detection dataset have demonstrated that integrating multi-head CNNs with LSTM networks yields superior performance over traditional HAR methods, particularly in healthcare applications [24]. Challenges such as computational intensity and reliance on multimodal data remain, but recent innovations in deep learning architectures have shown promise in addressing these issues [25]. A summary of recent HAR review studies and the remaining Gaps is shown in Table 1.

## 3. Benchmark Dataset

To compare deep learning and machine learning model employment on HAR, researchers have proposed various benchmark datasets [26]. Motion dataset contains motion signals of embedded wearable sensors of wearables or smartphone multi-sensors on body positions: chest, forearm, head, pocket, or wrist. Wearables can be worn on the head, shin, forearm, chest, upper arm, thigh, waist, and legs. If you like wearing your smartphone in your pocket and your smartwatch on your right wrist (because you are right-handed), you may do so. Sensors found in the datasets There are accelerometers (A), gyroscopes (G), magnetometers (M) and others (e.g., object, temperature, ambient) [26]. We have also recorded the ages and weights heights, etc., of the participants varied in each of the datasets: simple (walk, run, lie down), complex (cook, clean), and postural transitions (sit to stand). Detailed descriptions and information regarding the benchmark datasets are presented in Table 2 [27].

## 4. Feature Extraction and Machine Learning Based Approach

### 4.1. Multi-View HAR for AAL

The presence of such datasets such as the RHM dataset is strongly favorable for multi-view Human Activity Recognition (HAR). These datasets all record an enormously large variety of activities from multiple views so that the models can learn to recognize human activities in three-dimensional space. Multi-view data eliminates the limitations of single-view systems such as occlusion and perspective distortion and thus enhances the ability of the system to recognize complex activities [27]. Such data like the RHM dataset are an important contribution towards the creation of multi-view HAR. They capture diverse activities from many different views so that models can be trained better to represent human actions in 3D. Multi-view data can provide relief from single-view system limitations such as occlusion and perspective distortion for the best opportunity for accurate recognition of complex activities [54]. Through the exploitation of sensor data from different views and perspectives, multi-view HAR facilitates the ability of the system to identify activities more accurately. It covers different applications like environmental sensors, wearable sensors, and vision-based systems and targets the requirement for the unification of data sources across the applications. HAR system design in complex AAL environments comprises the combination of advanced methods like sensor fusion consisting of data-level, feature-level, and decision-level fusion.

**Figure 1:**
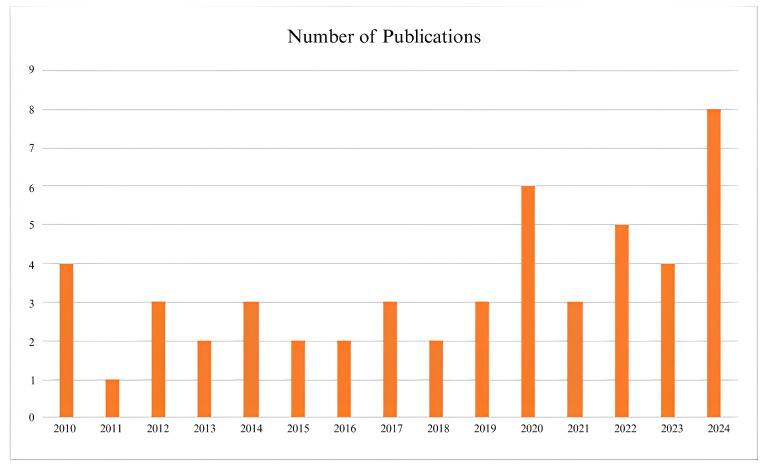
Year-wise peer-reviewed publications used in this study.

**Figure 2:**
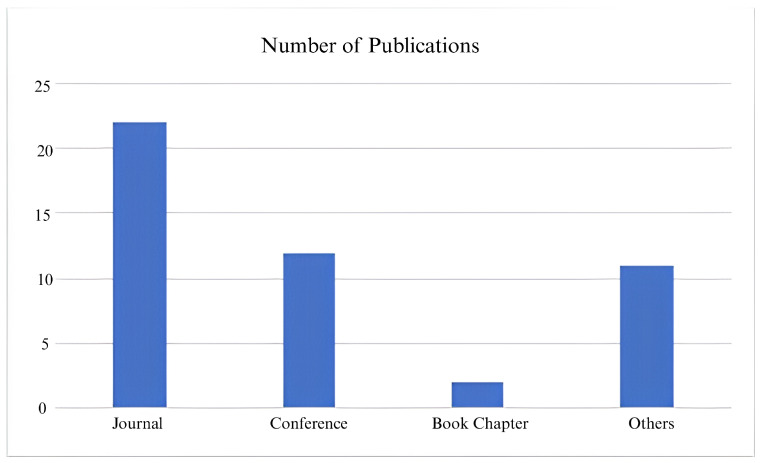
Articles type journal, conference, book chapters, and others.

Figure 1 presents the number of publications by year, highlighting a noticeable increase in research activity on Human Activity Recognition (HAR) in recent years, with a particularly sharp rise from 2020 onwards. The year 2024 recorded the highest number of publications, reflecting the growing academic focus on multi-view HAR systems and lightweight deep learning applications in Ambient Assisted Living (AAL). Complementing this, Figure 2 categorizes the selected publications by type, indicating that journal articles constitute the majority, followed by conference proceedings, book chapters, and other sources such as preprints and dataset reports. This distribution emphasizes the predominance of peer-reviewed journal publications in advancing HAR research while also demonstrating the valuable contributions from diverse publication platforms in this rapidly evolving field.

### 4.2. Feature Extraction in Human Activity Recognition

**Figure 3:**
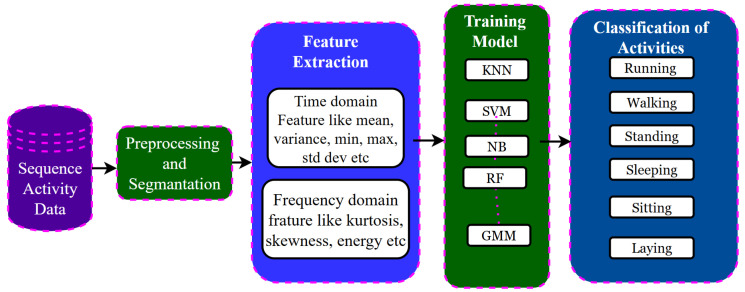
Human Activity Recognition using conventional ML-based techniques.

The data segments obtained from the previous step are used for feature extraction, the most important stage in the Human Activity Recognition (HAR) pipeline. Feature extraction may be manual or automatic. Machine learning-based methods adopt these pipelines of data acquisition, data preprocessing and segmentation, feature extraction with hand-crafted features, feature selection, and finally, classification [55] depicts in Figure 3.

### 4.3. Feature Learning

DL-based approaches can learn features automatically without any manual feature engineering.

## 5. Deep Learning Models

**Figure 4:**
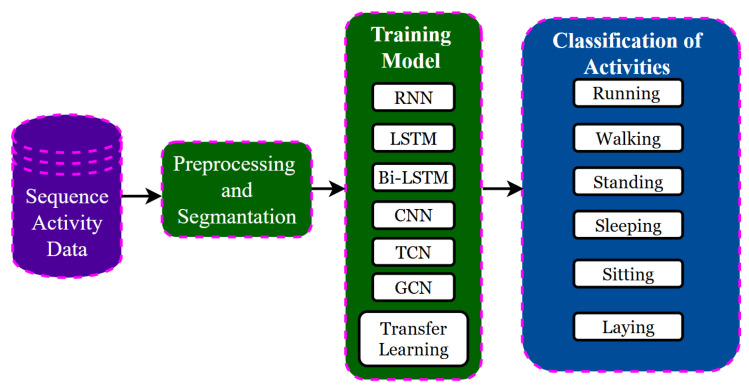
Flowchart of the DL based systems.

**Figure 5:**
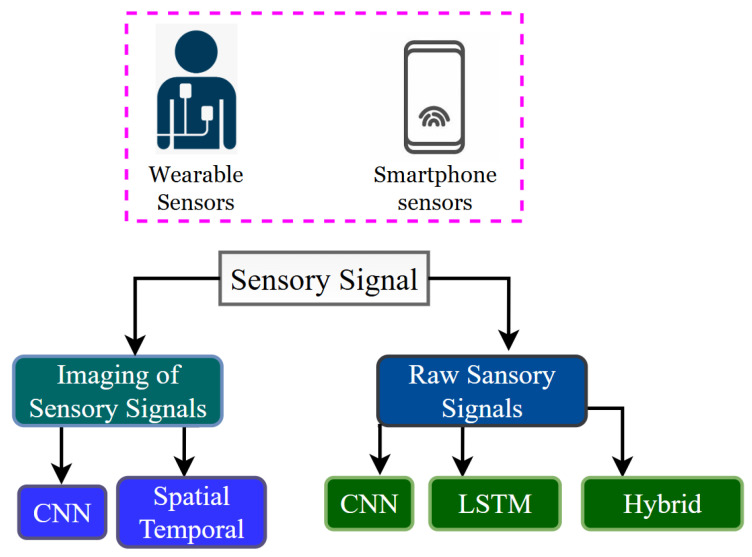
Categorization of the Deep learning model.

Machine learning has attracted researchers to explore deep learning models for Human Activity Recognition (HAR) due to their potential for high reward. Figure 4 illustrates the HAR using DL approaches for feature extraction and classification. These models are usually categorized based on the input data they receive [26]. Figure 5 illustrates the categorization of deep learning models based on the type of sensory signals acquired through wearable sensors and smartphones for Human Activity Recognition (HAR). The sensory data are classified into two main types: imaging of sensory signals and raw sensory signals. In the case of imaging, sensor data are transformed into visual representations such as spectrograms or encoded plots, which are then processed using Convolutional Neural Networks (CNNs) to extract spatial features. For raw sensory signals, the data are directly fed into models without transformation. CNNs are used to capture local dependencies, Long Short-Term Memory (LSTM) networks are applied to learn temporal patterns in sequential data, and hybrid models combine multiple architectures, such as CNN-LSTM combinations, to leverage both spatial and temporal features. This categorization highlights how the nature of the sensory input influences the choice of deep learning architecture, guiding the development of accurate and efficient HAR systems, particularly in resource-constrained Ambient Assisted Living (AAL) environments. The HAR pipeline can be simplified with deep learning (DL) methods. DL has been shown to achieve outstanding empirical performance in many applications such as image synthesis, image segmentation, image classification, object detection, activity recognition, and human gait analysis [56]. DL algorithms are composed of multiple abstraction levels constructed through the neurons. Feature maps are obtained from the input feature maps of the preceding layer through the non-linear functions of each layer. Hierarchical abstraction enables DL algorithms to learn one level-specific feature for each domain of application. Deep learning uses a Deep Neural Network (DNN) architecture where a specific loss function is optimized for feature extraction as well as determination of the classification boundaries [57].

### 5.1. DL Categories Based on Sensory Cues

On the basis of the imagery signal type it can be divided into the following two categories:

#### Imaging of Sensory Signals

Convolutional Neural Networks (CNNs) are most appropriate for extracting the features of images and classifying large amounts of image data. The technique is also effective for time-series analysis where sensor data are converted into time-series images and passed through the CNN. A smartphone-based platform was shown by Alemayoh et al., where raw sensor data are accepted and 14×60 virtual images are constructed. A CNN (1C-1P-2C-2P-1DL) is employed to classify images into eight activities [58].

Some of the multimodal approaches, such as that of Lawal et al., incorporate wearable sensor signals into frequency images. Their effectiveness with the fusion technique is demonstrated with their two-stream CNN model [59]. Qin et al. proposed heterogeneously processing sensor data, modeling the signals as images with two channels and propose residual fusion using the Lawal et al.’s layers [60]. It has been quantitatively evaluated and compared with state-of-the-art on the HHAR and MHEALTH datasets [61].

### 5.2. Raw Sensory Data

Deep learning methodologies with their local dependency modeling and preservation of scaling-invariance are of immense usefulness for feature learning and classification of time-series data. They are thus gaining increasing popularity for Human Activity Recognition systems. Interdisciplinary research on CNNs, LSTMs, and hybrid deep learning techniques for improving the performance of HAR systems is an active research field [62].

### 5.3. Multi-View HAR with CNN-Based Methods in AAL

Convolutional Neural Networks (CNNs) have played a major role in Human Activity Recognition (HAR) modeling due to their capability of efficiently classifying images as well as raw time-series data (low-level information) of various sensors. CNNs are efficient due to their capability of discovering local dependencies, scale-invariant features, and learning complicated non-linear relationships within the input data—desired characteristics making CNNs of great utility for HAR applications [63]. CNNs were utilized to transform raw sensor data for HAR. Novel strategies are founded upon novel CNN structures for sensor data types, i.e., tri-axial accelerometer data typically used in smartphones. Small pool sizes of convolutional layers are best suited for extracting and discriminating valuable features with better activity recognition accuracy [64,65]. There has also been research that has combined CNNs with statistical features to learn local as well as global features of the signal. A hybrid approach like this maintains detailed information of the signal at various scales. Multi-parallel CNN systems have been suggested for user-dependent activity recognition to emphasize local feature extraction and combination of features. However, CNNs are computationally demanding, something often referred to as “frugality”. This has led to efforts at optimization and hence efficient CNN models that are effective but less demanding. Techniques like Lego filters and Conditionally Parameterized Convolutions optimize the efficiency of the HAR process [66]. Also, applying pre-trained CNNs with dimensionality reduction like PCA and then hybrid classifiers like SVMs can optimize runtime—a major concern on smartphones where battery power is precious. Fair enough, CNNs are good at recognizing simple actions but discriminating more complex and related actions is not easy. Ensemble CNNs with different architecture are being suggested as the answer to make HAR systems more sensitive and specific [67]. Transfer learning schemes, for instance, the proposal of Heterogeneous Deep CNN (HDCNN) models, generalize across different sensor domains. Since HAR detectors are position-dependent on sensor placement, other studies have explored determinant-agnostic methods [68]. These systems attempt to work regardless of sensor placement on the body but lose accuracy at certain instances. Deep learning models are now being constructed to be sensor position robust with satisfactory performances on practical data. The “cold-start” problem or vanishing gradients in CNNs remains even now and has led to even more complex network architecture such as residual networks in CNNs. These are aimed towards more detailed activity-type recognition of varied dynamic activities for enhancing the applicability of HAR in Ambient Assisted Living (AAL) environments [69]. The future of HAR with CNNs in AAL environments requires continued research into architecture designs, hybrid models, and deep learning strategies. This will upgrade the perception, interaction, and functionality of HAR systems in AAL and promote the well-being and independence of users. Recent advancements in Human Activity Recognition (HAR) have seen the ubiquitous adoption of Convolutional Neural Networks (CNNs) for sensor data analysis of wearables and mobile devices. Ignatov et al. [70] employed a deep learning technique that takes advantage of CNNs for sensor data-based HAR in real-time. Their proposed technique aims at local feature extraction using CNNs with the assistance of simple-to-compute statistical features that detect global time-series patterns. They tested their technique using UCI and WISDM datasets with high accuracy for various users and datasets. This is proof of their deep learning technique being effective without requiring complex computational hardware and hand-crafted feature engineering. Taking advantage of the power of CNNs, Chen et al. [71] proposed the semi-supervised deep learning method optimized for imbalanced HAR using multimodal wearable sensor data. Their model addresses the shortage of fewer labeled samples and class imbalance using the pattern-balanced model that finds heterogenous patterns. With the recurrent convolutional attention networks, their model is capable of finding the most significant features across sensor modalities, which highly empowers the model to work in imbalanced data environments. Kaya et al. [72] proposed the 1D-CNN-based method for accurate HAR from sensor data with special focus on raw accelerometer and gyroscope sensor data. Their model was tested on three publicly released datasets—UCI-HAPT, WISDM, and PAMAP2—demonstrating the strength and flexibility of the model towards various data sources. The method simplifies feature extraction without accuracy degradation and thus is highly suitable for AAL applications in real-time. In another recent research work, Zhang et al. [73] proposed ConvTransformer, which is a novel method that combines CNNs, Transformer networks, and attention mechanisms to address the dual issue of extracting detailed and general features of sensor data. The hybrid method takes the power of CNNs in extracting local patterns while Transformers and attention mechanisms enable the model to learn global context, making it highly suitable for complex HAR in multi-view AAL environments.

### 5.4. RNN, LSTM, and Bi-LSTM in HAR for AAL

Recurrent Neural Networks (RNNs) have been at the core of recent Human Activity Recognition (HAR) studies due to their capacity in dealing with temporal dependencies in sensor data. For instance, Ordonez et al. [74] demonstrated how RNNs play a crucial role in capturing the sequentiality of sensor data, which is critical in guaranteeing activity recognition accuracy. However, traditional RNNs are prone to suffering from gradient vanishing issues, a problem that necessitated the development of Long Short-Term Memory (LSTM) networks [75]. LSTM networks retain information for longer sequences, thus addressing the issues brought about by long-range dependencies in HAR applications.

LSTMs are suitable for Human Activity Recognition (HAR) since they are capable of processing sequential time-series data without vanishing or exploding gradients by maintaining long-term dependencies, while CNNs excel at image recognition through discovering spatial correlations, LSTM models use feedback connections to discover time patterns. A review of LSTM-based HAR systems, challenges, and future research directions is given. Early studies on on-body sensor placements explored LSTM models, but most did not crowdsource enough data from a diverse range of individuals to generalize to more than a few basic activities. On the computation limitation side, Agarwal et al. proposed an edge-device-friendly lightweight LSTM model but did not test it on complex activities. Extending this idea, Rashid et al. focused on energy efficiency and sensor data processing [76]. To overcome the vanishing gradient issue, Zhao et al. suggested a residual bidirectional LSTM architecture that concatenates forward and backward states to improve performance. Their approach maintained high performance in the dynamic and complex setting of HAR [76]. In [74], Wang and Liu suggested a Hierarchical Deep Long-Short Term Memory (H-LSTM) model, reducing the interference of noise and making feature learning easier in time–frequency domains. Similarly, Ashry et al. suggested a cascaded LSTM model that fused raw signal information and feature-based information, reducing the need for large-scale training datasets.

To address the local feature analysis weaknesses in traditional HAR approaches, a new hybrid deep learning framework was proposed. It took advantage of the convolutional capability of CNNs (layer-wise) for feature extraction, while LSTM units processed sequential information. The LSTM performance was also enhanced with the use of Extreme Learning Machine (ELM) classifiers. On the other hand, Zhou et al. addressed the problem of insufficient sensor labels. Their approach was to train a semi-supervised LSTM model combined with a Deep Q-Network, which annotates data automatically, improving performance in noisy and well-labeled dataset settings.

Researchers have also explored the use of attention-based Bidirectional LSTM (Bi-LSTM) models to further improve the performance of HAR systems [77,78,79,80]. Such models have been shown to achieve superior performance compared to other deep learning-based methods, as validated by experimental comparisons on several benchmark datasets. The results, as indicated in Table 3, show that attention-based Bi-LSTM models can achieve high accuracy for different user groups and datasets, while requiring relatively modest computational resources and without large-scale manual feature engineering.

Saha et al. [95] suggested Fusion ActNet, a new method for HAR that uses sensor data to differentiate between static and dynamic actions. The model includes individual residual networks for both actions and a decision guidance module. The method is trained in two stages and has been thoroughly experimented upon using benchmark datasets, and it has been shown to be efficient in complex HAR scenarios, particularly in Ambient Assisted Living (AAL) environments where a wide range of activities have to be accurately detected. Murad et al. [75] have highlighted the merits of using Deep Recurrent Neural Networks (DRNNs) for HAR, specifically in capturing long-range dependencies in variable-length input sequences from body-worn sensors. Unlike traditional methods that fail to consider temporal correlations, DRNNs—unidirectional, bidirectional, and cascaded LSTM networks—yield state-of-the-art performance on several benchmark datasets. They compared DRNNs to traditional machine learning techniques like Support Vector Machines (SVM) and k-Nearest Neighbors (KNN) and other deep learning methods like Deep Belief Networks (DBNs) and CNNs, consistently demonstrating the superior performance of DRNNs in Human Activity Recognition.

### 5.5. Integration of CNN and LSTM-Based Techniques

In recent years, there has been significant progress in the development of hybrid deep learning models that combine different architectures to achieve high-performance Human Activity Recognition (HAR). One popular approach involves integrating Convolutional Neural Networks (CNNs) with Long Short-Term Memory (LSTM) networks to leverage the strengths of both models. For example, hybrid CNN-LSTM models have been found to achieve superior performance in various applications, including sleep–wake detection using heterogeneous sensors [88,96]. These models benefit from the ability of CNNs to extract local spatial features from sensor data and the capability of LSTMs to capture temporal dependencies, making them particularly effective in HAR applications where both spatial and temporal information are crucial.

Another notable design is the TCCSNet, which, along with CSNet, enhances human behavior detection and recognition by leveraging both temporal and channel dependencies [97]. These architectures effectively combine the strengths of CNNs and LSTMs, enabling more accurate recognition of complex human activities by modeling both spatial and temporal aspects of the data. Ordóñez et al. further explored this integration by developing a CNN-LSTM-based model for HAR [74]. Their approach involves feature extraction from raw sensor data using CNNs, followed by processing with LSTM recurrent units to model complex temporal dynamics. This model also supports multimodal sensor fusion, allowing it to handle data from multiple sensor types without requiring manual feature design. Evaluation on benchmark datasets such as Opportunity and Skoda revealed significant performance improvements over traditional methods, underscoring the effectiveness of this hybrid approach in HAR applications.

Zhang et al. introduced a multi-channel deep learning network called the 1DCNN-Att-BiLSTM hybrid model, which combines 1D CNNs with attention mechanisms and Bidirectional LSTM units [80]. The model was evaluated using publicly available datasets, and the performance metrics demonstrated improved recognition accuracy compared to both conventional machine learning and deep learning models. The hybrid approach of integrating CNNs, LSTMs, and attention mechanisms provides a promising framework for handling the complexities of multi-view HAR in Ambient Assisted Living (AAL) settings.

El-Adawi et al. developed a HAR model within the context of Wireless Body Area Networks (WBAN), utilizing a novel approach that integrates the Gramian Angular Field (GAF) transformation with DenseNet [91]. In this model, time-series data are converted into 2D images using the GAF technique and subsequently processed by DenseNet to achieve high-performance accuracy. This innovative method demonstrates the potential for combining different deep learning techniques and data transformation strategies to enhance HAR, particularly in environments like AAL, where sensor data from body-worn wearable devices are prevalent.

### 5.6. TCN Approaches for Multi-View Human Activity Recognition in Ambient Assisted Living

Recent advancements in Human Activity Recognition (HAR), particularly within the context of Ambient Assisted Living (AAL), have increasingly focused on leveraging deep learning models to process and analyze data collected from diverse sensors. These sensors, which can be categorized as user-driven, environment-driven, or object-driven, play a crucial role in capturing the intricacies of human activity across different contexts [98]. Traditional methods have struggled to effectively capture the temporal dependencies inherent in sensor-driven time-series data. However, recent progress in deep learning, especially through the use of Transformer models founded on multi-head attention mechanisms, has significantly enhanced the ability to model these temporal relationships [99]. This capability is essential for accurately identifying and predicting activities in dynamic AAL environments, where the activities of interest may frequently change over time.

One of the most significant challenges in HAR is adapting systems to recognize new activities in ever-changing environments. To address this, researchers have emphasized the importance of incorporating sensor frequency information and conducting thorough analyses of both time and frequency domains. This approach not only improves the understanding of sensor-driven time-series data but also facilitates the development of more robust and efficient HAR systems [100]. In response to these challenges, Kim et al. [98] introduced a Contrastive Learning-based Novelty Detection (CLAN) method for HAR that utilizes sensor data. The CLAN method is particularly effective at handling temporal and frequency features, as well as the complex dynamics of activities and variations in sensor modalities. By leveraging data augmentation to create diversified negative pairs, this method enhances the system’s ability to detect novel activities even when they share features with known activities. The two-tower model employed by CLAN extracts invariant representations of known activities, improving the system’s ability to recognize new activities with similar characteristics.

Building on these concepts, Wei et al. [90] proposed a Time Convolutional Network with an Attention Mechanism (TCN-Attention-HAR) specifically designed to optimize HAR using wearable sensor data. The TCN-Attention-HAR model addresses critical challenges in temporal feature extraction and the gradient issues commonly encountered in deep networks. By optimizing temporal convolution sizes and incorporating attention mechanisms, the model effectively prioritizes important information, resulting in more accurate activity recognition. This approach is particularly advantageous in AAL scenarios, where real-time and lightweight processing is essential.

Zhang et al. [79] further advanced the field with the development of Multi-STMT, a multilevel model that combines spatiotemporal attention and multiscale temporal embedding to enhance HAR using wearable sensors. The Multi-STMT model integrates Convolutional Neural Networks (CNNs) with Bidirectional Gated Recurrent Units (BiGRUs) and attention mechanisms to capture subtle differences between human activities. This integration enables the model to accurately distinguish between similar activities, making it particularly valuable in multi-view HAR applications within AAL environments. Additionally, the Conditional Variational Autoencoder with Universal Sequence Mapping (CVAE-USM) proposed by Zhang et al. addresses the challenge of non-independent and identically distributed (non-i.i.d.) data in cross-user scenarios. By leveraging temporal relationships within time-series data and combining Variational Autoencoder (VAE) with Universal Sequence Mapping (USM) techniques, CVAE-USM effectively aligns user data distributions and extracts common temporal patterns across different users. This approach significantly enhances the accuracy of activity recognition, especially in multi-view AAL settings where between-user data variability remains a major concern.

#### TCN-Based Methods in Multi-View HAR for AAL

The adoption of Temporal Convolutional Networks (TCNs) in multi-view HAR for AAL environments offer unique advantages, particularly in terms of efficient temporal modeling. TCNs are specifically designed to handle sequential data by applying convolutional operations along the temporal dimension. This structure allows TCNs to effectively capture long-range dependencies and patterns in activity sequences, which are crucial for accurate recognition in multi-view settings. Moreover, the integration of attention mechanisms within TCN frameworks, as seen in the TCN-Attention-HAR model, allows for dynamic weighting of important temporal features, ensuring that the most relevant information is emphasized during the recognition process. This capability is particularly beneficial in AAL environments, where real-time decision-making is critical, and the system must be lightweight enough to operate efficiently on limited hardware.

The combination of TCNs with other deep learning models, such as CNNs and BiGRUs, further enhances their effectiveness in multi-view HAR. These hybrid models, like the Multi-STMT, can simultaneously capture spatial and temporal dependencies, providing a comprehensive understanding of human activities across different views. This multi-view capability is essential in AAL environments, where activities may be observed from various angles and perspectives.

In summary, the integration of TCN-based methods into multi-view HAR systems for AAL represents a significant advancement in the field. These methods not only improve the accuracy and efficiency of activity recognition but also ensure that the systems remain lightweight and responsive—key requirements for real-world applications in Ambient Assisted Living.

### 5.7. GCN-Based Multi-View HAR for AAL

Graph Convolutional Neural Networks (GCNNs) have emerged as a crucial tool in the field of Human Action Recognition (HAR), especially within the context of multi-view analysis for Ambient Assisted Living (AAL) environments. Unlike traditional deep learning models like Convolutional Neural Networks (CNNs) and Recurrent Neural Networks (RNNs), which are designed to handle Euclidean data structures such as images, text, and sequential data, GCNNs excel at processing non-Euclidean data where the relationships between data points are more complex and graph-structured. This unique capability makes GCNNs particularly well-suited for HAR in AAL settings, where understanding the nuanced movements of individuals across multiple views is critical.

#### 5.7.1. The Role of GCNs in Multi-View HAR

GCNs, first introduced by Franco Scarselli, have since evolved into a powerful framework for learning from graph-structured data [101]. In the context of HAR, human skeleton data, which consists of joint coordinates and their interconnections, can be naturally represented as a graph. Each joint is modeled as a node, while the connections between joints (e.g., bones) are treated as edges. This graph-based representation enables GCNNs to capture the intricate spatial dependencies between joints, which is essential for recognizing complex actions, especially when viewed from multiple angles.

In multi-view HAR, the challenge is further compounded by the need to integrate information from different perspectives. GCNs are uniquely positioned to address this challenge due to their ability to aggregate and process information across multiple views, ensuring that the resulting models can effectively generalize across varying conditions typical of AAL environments.

#### 5.7.2. Spectral GCN-Based Methods

Spectral GCNs leverage the principles of spectral graph theory, utilizing the eigenvalues and eigenvectors of the Graph Laplacian Matrix (GLM) to transform graph data from the spatial domain into the spectral domain [102]. This transformation facilitates the application of convolutional operations on graph-structured data. However, while effective, spectral GCNs have historically faced limitations in computational efficiency—a significant consideration in lightweight models for AAL applications. Kipf et al. [103] addressed this limitation by introducing a simplified version of spectral GCNs, where the filter operation is constrained to only one-hop neighbors. This enhancement significantly reduces computational overhead, making spectral GCNs more feasible for real-time HAR applications, including those required in AAL settings. Nevertheless, the fixed nature of the graph structure in spectral GCNs can be a limitation when dealing with the dynamic and variable nature of human actions viewed from multiple perspectives.

#### 5.7.3. Spatial GCN-Based Methods

Spatial GCNs have become the focal point of research in GCN-based HAR, particularly for multi-view scenarios. Unlike spectral GCNs, spatial GCNs operate directly on the graph in the spatial domain, allowing for more intuitive and flexible modeling of dynamic human actions. This flexibility is especially crucial in AAL environments, where actions must be recognized accurately across different views and conditions.

A significant advancement in this domain was the introduction of the Spatio-Temporal Graph Convolutional Network (ST-GCN) by Yan et al. [104]. The ST-GCN model is designed to handle both spatial and temporal aspects of human motion, capturing the evolving relationships between joints over time. In the context of multi-view HAR, ST-GCNs can effectively integrate information from various viewpoints, providing a holistic understanding of the action being performed.

Further enhancing the flexibility of GCNs in multi-view HAR, Shi et al. [105] developed the Two-Stream Adaptive Graph Convolutional Network (2s-AGCN). This model introduces an adaptive mechanism that allows the network to learn the graph topology dynamically, making it highly adaptable to diverse datasets and varying viewpoints typical in AAL settings. The inclusion of an attention mechanism further improves the model’s ability to focus on the most critical joints and their connections, ensuring robustness in action recognition across multiple views.

Shiraki et al. [106] advanced this concept with the Spatiotemporal Attentional Graph Convolutional Network (STA-GCN), which specifically addresses the varying importance of joints in different actions. STA-GCN not only considers the spatial relationships but also the temporal significance of joints, making it particularly effective for multi-view HAR where the importance of certain joints may vary depending on the action and the viewpoint.

#### 5.7.4. Recent Innovations of GCN

Recent innovations, such as the Shift-GCN model introduced by Shi et al. [107], have pushed the boundaries of multi-view HAR further by expanding the receptive field of spatiotemporal graphs and employing lightweight techniques to reduce computational costs [108]. This approach aligns well with the goals of AAL, where lightweight models must balance efficiency and accuracy. Other approaches, such as the Partial-Based Graph Convolutional Network (PB-GCN) developed by Thakkar et al. [109] and Li et al. [82], focus on segmenting the human skeleton into distinct parts, allowing the network to learn more focused and specialized representations of human actions. These methods are particularly useful in multi-view HAR, where the ability to isolate and analyze specific body parts can lead to more accurate recognition of complex actions.

The use of GCN-based methods in multi-view HAR for AAL is unique due to their ability to capture and integrate spatial-temporal dependencies from multiple perspectives. These methods allow for the development of lightweight, yet highly accurate models that are essential for real-time applications in AAL. The ongoing advancements in spatial GCNs, particularly with the integration of attention mechanisms and adaptive learning techniques, are setting new standards in the field, enabling more robust and flexible human action recognition systems. By leveraging these innovations, your survey paper aims to highlight the cutting-edge developments in GCN-based multi-view HAR, offering insights into how these methods can be optimized for AAL applications. This focus on lightweight, efficient models makes your work particularly relevant for practical implementations, ensuring that the systems are both scalable and effective in real-world AAL environments. In summary, the unique advantages of GCN-based methods, particularly the flexibility and efficiency offered by spatial GCNs, make them highly suitable for multi-view Human Activity Recognition in Ambient Assisted Living scenarios. By continuously refining these models and integrating innovative techniques like attention mechanisms and residual connections, researchers are pushing the boundaries of what is possible in HAR, making systems more accurate, adaptable, and efficient.

### 5.8. Transfer Learning-Based Lightweight Deep Learning

Traditional deep learning models are computationally intensive and challenging to deploy in real-time edge-based Ambient Assisted Living (AAL) systems due to their high computational demands. The previous chapter discussed the architecture and functioning of lightweight models such as MobileNet, SqueezeNet, and EfficientNet, along with approaches to reduce computational requirements, including depth-wise separable convolutions and squeeze-and-excitation blocks [110,111,112]. Furthermore, model compression and quantization techniques are reviewed to enhance the deployability of deep learning models in AAL systems [113].

Many researchers have introduced lightweight deep learning models that balance computational efficiency and model performance. These models, optimized for real-time processing, are crucial for AAL applications, where precise and fast activity recognition is essential [114,115]. Techniques such as model pruning, quantization, and knowledge distillation enable the derivation of compact and efficient models without significantly compromising accuracy [116,117].

This section focuses on the evolution of multi-view Human Activity Recognition (MV-HAR) beyond traditional methodologies and explores how integrating multiple viewpoints enhances performance. By leveraging multiple sensors or cameras positioned at different angles, MV-HAR improves visibility and ensures highly accurate activity detection [104]. The significance of energy-efficient processing in real-time applications, such as mobile devices and large-scale smart environments, is underscored. In the context of assistive living, personalized service options and proactive care strategies play a crucial role [102,118].

By integrating multiple camera perspectives, we demonstrate significant improvements in recognition accuracy, offering a scalable and practical solution for AAL systems with constrained computational resources. This initiative aims to enhance the quality and customization of support services for residents, including fall detection and home security. Leveraging advancements in machine learning, the proposed approach promotes safer and more comfortable living environments.

#### Resource Efficiency with Lightweight Deep Learning

Deep learning models scale out at the expense of higher computational complexity and storage. To mitigate these and improve model size and speed, lightweight deep learning techniques have been developed, including model compression, pruning, quantization, and knowledge distillation. These techniques aim for smaller and faster models with as minimal compromises in accuracy as possible and are thus suitable for power-constrained devices typical of most AAL settings.

## 6. Problems, Challenges, and Current/Future Directions

Although multi-view Human Activity Recognition (HAR) has made significant advances, it is still faced with many challenges, such as data synchronization, privacy protection, and the need for universal models. Solving these challenges will require new solutions, such as new synchronization techniques, secure data processing, and adaptive models—models that do not require large amounts of data to train and can simply adapt to changes in the environment. With the rapid development of micro-electromechanical systems and sensor technology, the recognition task of HAR has gained increasing attention in recent years. It is a crucial problem for a variety of applications such as Ambient Assisted Living, smart homes, sports, and work injury detection. HAR is a new field where recent developments driven by deep learning have opened up new possibilities. However, these solutions are still limited by problems that restrict their practical applicability. Table 4 shows the summary of challenges and future enhancements in HAR.

Labeled data are a precious commodity in NLP and one of the largest bottlenecks in HAR. Deep learning models require vast quantities of labeled data to train and test. However, acquiring and labeling these data are a time-consuming and costly process. This shortage of data can lead to models overfitting and failing to generalize well to real-world situations [119]. HAR models perform poorly in outdoor environments due to parameter variation when counting specific movements, i.e., push-ups and squats. Most HAR models are trained and tested in controlled laboratory environments. Outdoor environments are uncontrollable and contain several dozen factors—i.e., light, noise, and weather—that greatly affect sensor data. Therefore, high-performance HAR models in controlled environments may not offer the same accuracy outdoors. In addition, HAR models are challenged when they have to handle complex activities and postural transitions. Most HAR tasks consider simple activities such as walking, sitting, and sleeping. Daily activities also involve cooking, cleaning, and dressing. Moreover, HAR models are not effective in identifying falls and other postural transitions. One of the pitfalls involves the need for meticulous hyperparameter tuning in order to realize the best model performance. Hyperparameters are settings of learning algorithms that one may adjust to derive optimum results. However, the vast number of hyperparameters can render it challenging to determine the most suitable settings for any problem. One other challenge is presented by the fusion of multimodal sensor data. Most HAR systems utilize only one type of sensor, e.g., an accelerometer. However, fusion of data from different sensors—e.g., gyroscopes and magnetometers—can enhance the performance of HAR. This leads to the challenge of how to fuse data from such heterogeneous sources optimally. The final limitation of HAR models is with regard to their expected performance in unsupervised learning. Supervised machine learning requires human labeling, while clustering is akin to supervised learning without the human element. Unsupervised learning differs from supervised learning because the latter requires labeled data, which is typically expensive and time-consuming to create. Yet, the majority of unsupervised HAR models are inferior to even the poorer-performing supervised models. In summary, there are some challenges for HAR that need to be considered to advance the field and improve its applications. Addressing these challenges will enable researchers to develop more precise and suitable HAR models for various environments. Although MV-HAR, when combined with lightweight models, holds promise for Ambient Assisted Living (AAL), there remain several challenges to be tackled, including

- Limited Multi-view AAL Datasets:
  There is a shortage of multi-view datasets for AAL, making it difficult to develop and compare models.
- Real-World Deployment: Privacy concerns, environmental robustness, and typical real-world AAL settings must be taken into account.

In summary, HAR for AAL using data acquired from diverse angles and leveraging low-cost deep learning approaches has a promising future. New directions such as sensor fusion, transfer learning, and latent domain adaptation offer the potential for enhancing HAR systems. To solve these issues, the research community must adopt an interdisciplinary approach to developing systems that are not just accurate but also privacy-compliant and adaptable to individual needs. According to the insights provided in these studies, this review will synthesize the knowledge that is available, enlighten the gaps that are present, and provide future research directions.

## 7. Conclusions

The lightweight deep learning approach referenced in this study effectively integrates multi-view Human Activity Recognition (HAR), showing strong potential in enhancing accuracy and efficiency within Ambient Assisted Living (AAL) applications. Significant advancements have been made from 2014 to 2024 in vision-based, sensor-based, and multimodal Hand Gesture Recognition (HGR) systems, particularly in data acquisition, gesture portrayal, and adaptable environments (RQ1). Various data modality-based HGR systems have demonstrated notable effectiveness, with emerging trends pointing toward increased adaptability, personalization, and real-world deployment potential (RQ2). However, key challenges such as modality-specific limitations, system usability, and real-time performance continue to impact scalability and user adoption (RQ3). Promising directions for future research include the design of more lightweight architectures, transfer learning for personalized HAR, development of privacy-preserving mechanisms, and the construction of large-scale, multi-view datasets to ensure inclusivity, especially for elderly and disabled populations in need of dependable AAL solutions.

## Figures and Tables

**Table 1:** Summary of Recent HAR review studies and remaining gaps.

Study	Focus Area	Key Contributions	Closed Gaps	Remaining Gaps	Uniqueness of This Manuscript
Guerra et al. (2023) [24]	HAR in AAL	Overview of AAL use cases and challenges	Application-level gap in frailty/fall detection	Lacks technical comparison of ML/DL and multi-view integration	Detailed comparison of SV vs. MV HAR with DL model evaluations
Chen et al. (2021) [18]	Sensor-based DL HAR	Survey of DL architectures for HAR	Identified scalability and accuracy issues	No focus on multi-view systems or lightweight models	Emphasizes real-time MV-HAR models for AAL
Liu et al. (2020) [22]	HAR Benchmarks	Dataset evaluations (e.g., NTU RGB+D)	Standardized benchmarks for HAR research	No application in AAL or focus on sensor fusion	Contextualized benchmark datasets for MV and AAL systems
Yang et al. (2020) [23]	Skeleton-based HAR (GCN)	Graph-based feature learning using GCNs	Improved spatio-temporal graph modeling	No comparison of SV vs. MV or deployment context	Combines CNN, GCN, TCN for multi-perspective HAR in AAL
Duan et al. (2022) [25]	Skeleton Action Recognition	Review of ST-GCN advances	Enhanced temporal pattern modeling	Limited focus on privacy, real-world deployment	Includes privacy-preserving and lightweight DL in MV-HAR

**Table 2:** List of publicly available HAR for AAL related benchmark datasets.

Dataset Name	Number of Activities	Activity Type	Number of Subjects	Sampling Frequency	Devices Used	Device Position	Latest Performances
PAM-AP2 [28]	12	ADLs, Postural, Complex	9	100 Hz	3 IMU units, 1 Heart rate monitor	Wrist, chest, dominant ankle	
HHAR [29]	6	ADLs	9	Highest Available	Smartwatches, Smartphones	Smartphone on waist, pouch	
MHEA-LTH [30]	12	ADLs	10	50 Hz	Shimmer2 sensors	Right wrist, left ankle, chest	
UCI-HAR [31]	6	ADLs	30	50 Hz	Smartphone	Left belt, no specific position	
OPPOR-TUNITY [32]	6	ADLs, Complex	4	-	Body-worn, Object, Ambient Sensors	Upper body, hip, leg, shoes	
WISDM [33]	18	ADLs, Postural, Complex	51	20 Hz	Smartphone, Smartwatch	Right pant pocket, dominant hand (watch)	
UniMiB-SHAR [34]	17	ADLs, Falls	30	50 Hz	Smartphone	Left right trouser pockets	
Mobi-Act [35]	16	ADLs, Falls	66	100 Hz	Smartphone	Trouser pocket	
HHAR [29]	6	Daily living activity, Sports fitness activity	9	Highest Available	Accelerometer, Gyroscope	Smartphone on waist, pouch	99.99%
MHEA-LTH [36]	12	Atomic activity, Daily living activity, Sports fitness activity	10	50 Hz	Accelerometer, Gyroscope, Magnetometer, Electrocardiogram	Right wrist, left ankle, chest	97.83%
OPPOR-TUNITY [37]	17	Daily living activity, Composite activity	4	-	Acceleration, Rate of Turn, Magnetic field, Reed switches	Upper body, hip, leg, shoes	100%
WISDM [38]	6	Daily living activity, Sports fitness activity	33	20 Hz	Accelerometer, Gyroscope	Right pant pocket, dominant hand (watch)	97.8%
UCI-HAR [39]	6	Daily living activity	30	50 Hz	Accelerometer, Gyroscope	Left belt, no specific position	95.90%
PAMA-P2 [40]	18	Daily living activity, Sports fitness activity, Composite activity	9	100 Hz	Accelerometer, Gyroscope, Magnetometer, Temperature	Wrist, chest, dominant ankle	94.72%, 82.12%, 90.27%
DSADS [41]	19	Daily living activity, Sports fitness activity	8	45 Hz	Accelerometer, Gyroscope, Magnetometer	-	99.48%
Real-World [42]	8	Daily living activity, Sports fitness activity	15	7	Acceleration	-	95%
Exer. Activity [43]	10	Sports fitness activity	20	-	Accelerometer, Gyroscope	-	-
UTD-MHAD [44]	27	Daily living activity, Sports fitness activity, Composite activity, Atomic activity	8	-	Accelerometer, Gyroscope, RGB camera, depth camera	-	76.35%
TUD [45]	34	Daily living activity, Sports fitness, Composite activity	1	-	Accelerometer	-	-
UCI HAR and UniMiB SHAR [46]	17	Daily living activity, Sports fitness activity, Atomic activity	30	-	Accelerometer	Left right trouser pockets	82.79%
USC-HAD [47]	12	Daily living activity, Sports fitness activity	14	-	Accelerometer, Gyroscope	-	97.25%
Mobi-Act [48]	13	Daily living activity, Atomic activity	50	100 Hz	Accelerometer, Gyroscope, Orientation sensors	Trouser pocket	75.87%
Motion Sense [49]	6	Daily living activity	24	50 Hz	Accelerometer, Gyroscope	-	95.35%
CASAS [50]	7	Daily living activity, Composite activity	1	-	Temperature, Infrared motion/light sensor	-	88.4%
Skoda [51]	10	Daily living activity, Composite activity	1	-	Accelerometer	-	97%
Wida-r3.0 [52]	6	Atomic activity	1	-	Wi-Fi	-	82.18%
HAPT [53]	12	Human activity	30	50 Hz	Accelerometer, Gyroscope	-	92.14%, 98.73%

**Table 3:** Sensor data modality-based HAR models, performance, and problem-solving approaches.

Author	Year	Dataset Name	Modality Sensor Name	Methods	Classifier	Accu Racy %	Ways to Solve the Problem
Ignatov et al. [70]	2018	WISDM, UCI HAR	IMU	CNN	SoftMax	93.32, 97.63	Enhance spatial feature extraction from IMU data
Jain et al. [81]	2018	UCI HAR	IMU	Fusion-based	SVM, KNN	97.12	Combine multiple classifiers for improved decision boundaries
Chen et al. [71]	2019	MHEALTH, PAMAP2, UCI HAR	IMU	CNN	SoftMax	94.05, 83.42, 81.32	Improve spatial feature learning, reduce manual feature design
Alawneh et al. [77]	2020	UniMib Shar, WISDM	Accelerometer, IMU	Bi-LSTM	SoftMax	99.25, 98.11	Capture long-term temporal dependencies for sequential activities
Lin et al. [82]	2020	Smartwatch	Accelerometer, Gyroscope	Dilated CNN	SoftMax	95.49	Expand receptive fields without increasing parameters for temporal feature capture
Zhang et al. [80]	2020	WaFi CSI	WiFi Signal	Dense-LSTM	SoftMax	90.0	Enhance sequence modeling in WiFi-based HAR
Nadeem et al. [83]	2021	WISDM, PAMAP2, USC-HAD	IMU	HMM	SoftMax	91.28, 91.73, 90.19	Handle sequential state transitions and temporal uncertainty
Kavunc_ uouglu et al. [84]	2021	Fall and ADLs	Accelerometer, Gyroscope, Magnetometer	ML	SVM, KNN	99.96, 95.27	Evaluate sensor heterogeneity and optimize classifier performance
Lu et al. [85]	2022	WISDM, PAMAP2, UCI-HAR	IMU, Accelerometers	CNN-GRU	SoftMax	96.41, 96.25, 96.67	Combine spatial and sequential feature extraction
Kim et al. [86]	2022	WISDM, USC-HAR	IMU	CNN-BiGRU	SoftMax	99.49, 88.31	Handle imbalanced data with oversampling and enhance temporal modeling
Sarkar et al. [87]	2023	UCI-HAR, WISDM, MHEALTH, PAMAP2, HHAR	IMU, Accelerometers	CNN with GA	SVM	98.74, 98.34, 99.72, 97.55, 96.87	Optimize feature selection and improve classifier robustness
Semwal et al. [88]	2023	WISDM, PAMAP2, USC-HAD	IMU	CNN and LSTM	SoftMax	95.76, 94.64, 89.83	Integrate spatial and sequential feature extraction
Yao et al. [89]	2024	PAMAP2, USC-HAD, UniMiB-SHAR, OPPORTUNITY	IMU, Accelerometers	ELK ResNet	SoftMax	95.53, 97.25, 82.79, 87.96	Leverage residual learning for deep spatial feature extraction
Wei et al. [90]	2024	WISDM, PAMAP2, USC-HAD	IMU	TCN-Attention	SoftMax	99.03, 98.35, 96.32	Model long-term temporal features with attention prioritization
El-Adawi et al. [91]	2024	MHEALTH	IMU	GAF+ DenseNet169	SoftMax	97.83	Convert time-series to images for spatial feature extraction
Ye et al. [92]	2024	OPPT, PAMAP2	IMU	CVAE-USM	GMM	100, 82.12	Handle cross-user non-i.i.d data with generative modeling
Kaya et al. [72]	2024	UCI-HAPT, WISDM, PAMAP2	IMU	Deep CNN	SoftMax	98, 97.8, 90.27	Extract complex spatial patterns from raw sensor data
Zhang et al. [93]	2024	Shoaib, SisFall, HCIHAR, KU-HAR	IMU	1DCNN-Att-BiLSTM	SVM	99.48, 91.85, 96.67, 97.99	Combine attention with deep sequential modeling for HAR
Zhang et al. [79]	2024	DSADS, HAPT	IMU	Multi-STMT	SoftMax	99.86, 98.73	Enhance multi-scale temporal feature extraction
Saha et al. [94]	2024	UCI HAR, Motion-Sense	IMU	Fusion_ ActNet	SoftMax	97.35, 95.35	Integrate multi-sensor data for improved activity classification

**Table 4:** Summary of challenges and future enhancements in HAR.

Challenge	Description
Scarcity of annotated data	Deep learning models are trained and evaluated on labelled data. However, all sensory data need to be collected and labelled, which makes it costly and time-consuming.
Limited performance in outdoor environments	HAR models are known to have poor performance in outdoor environments due to variable lighting, noise, and different weather conditions.
Difficulty recognizing complex activities and postural transitions	Simple activities and postural transitions may not be properly recognized by many HAR models.
Hyperparameter optimization	HAR models can particularly affect accuracy if the requisite hyperparameters are not properly tuned.
Fusion of multimodal sensor data	By fusing data from different sensors, data of various formats and resolutions can be combined to enhance the information available to HAR systems.
Performance limitations in unsupervised learning	One of the most common problems with unsupervised HAR models is their accuracy compared to supervised models.

## Data Availability

The original contributions presented in this study are included in the article. Further inquiries can be directed to the corresponding authors.

## Abbreviations

LSTM	Long-Short-Term-Memory
BiLSTM	BiDirectional long short-term Memory
CNN	Convolutional Neural Networks
GMM	Gaussian Mixture Model
DD-Net	Double-feature Double-motion Network
GAN	Generative Adversarial Network
SOTA	State-of-the-Art
MobileNetV2	Mobile Network Variant 2
ReLU	Rectified Linear Unit
DCNN	Dilated Convolutional Neural Network
GRNN	General regression neural network
KFDI	Key Frames Dynamic Image (
QST	Quaternion Spatial-Temporale
RNNs	Recurrent Neural Networks
BT-LSTM	Block-Term Long-Short-Memory
KF	Kalman filter
KM-Model	Keypoint mesh model
SRGB-Model	Segmented RGB model
